# Is the serotonin hypothesis dead? If so, how will clinical psychology respond?

**DOI:** 10.3389/fpsyg.2022.1027375

**Published:** 2022-11-03

**Authors:** Nicholas C. Borgogna, Stephen L. Aita

**Affiliations:** ^1^Department of Psychological Sciences, Texas Tech University, Lubbock, TX, United States; ^2^Veterans Affairs Maine Healthcare System, Augusta, ME, United States

**Keywords:** serotonin hypothesis, affective disorders, depression, clinical psychologists, medication

For decades models of mental illness, particularly depression, have been influenced by the serotonin hypothesis (Coppen, 1967; Fakhoury, 2016). Specifically, that dysregulation in the serotonin neural system is an underlying biological cause of affective disorders. This model is the primary justification for the prescription of selective serotonin reuptake inhibitors (SSRIs; e.g., Prozac [Fluoxetine], Zoloft [Sertraline], Lexapro [Escitalopram]). SSRI prescription is extremely popular within modern psychiatry, with an estimated 13% of people living in the United States having taken an SSRI within the past 30 days (Brody and Gu, 2020). World-wide estimates are difficult to obtain, but developed European nations report commensurate prescribing activity (Abbing-Karahagopian et al., 2014), with indicators suggesting that SSRI prescriptions are increasing worldwide (Lockhart and Guthrie, 2011; Chen et al., 2022). Notably, estimates suggest the SSRI industry to be worth over $15 billion (USD) as of 2021 (Antidepressants Global Market Report 2021: COVID-19 Implications Growth to 2030, 2021).

Despite the popularity of SSRIs, there have been increasing concerns about their safety, efficacy, and the validity of their mechanisms of change. Notably, SSRIs are associated with a variety of side-effects that can be particularly severe, such as suicidal thoughts (Whittington et al., 2004; Cascade et al., 2009; Locher et al., 2017; Wang et al., 2018). Previous meta-analytic findings have demonstrated that many SSRIs are no more effective than placebo, with the ones demonstrating significant efficacy only doing so with small effect sizes (Hetrick et al., 2010; Locher et al., 2017; Cipriani et al., 2018). Some researchers have even raised the possibility that the therapeutic effects of SSRIs are totally placebo (Cuijpers and Cristea, 2015), attributing significant therapeutic effects in SSRI conditions to methodological problems (such as blind penetration). Guidelines for prescription practices are also concerningly inadequate, especially for those who want to discontinue SSRI intervention (MacQueen et al., 2017; Sørensen et al., 2022). Finally, there has been compounding evidence that increasing serotonin *via* re-uptake inhibition (i.e., the mechanism of change) is not actually associated with any improvement in depression.

This point has most recently been made by Moncrieff et al. (2022) whose umbrella review (including multiple meta analyses) all but damns the serotonin hypothesis. Specifically, Moncrieff et al. (2022) offer compelling data that there is no consistent evidence linking lowered serotonin concentration/activity to depression. They suggest that it is “time to acknowledge the serotonin theory of depression is not empirically substantiated” (p. 12). Ironically, their review additionally suggests evidence that long-term use of SSRI's might actually reduce serotonin concentrations in the body.

While the prescription of psychoactive medications has largely been relegated to the field of psychiatry, clinical psychologists still play an important role in prescription facilitation. Psychologists often refer clients to psychiatrists or primary care physicians for SSRI prescriptions. Psychologists are integral in monitoring SSRI effectiveness and safety. Psychologists also often consult with prescribing physicians regarding potential medication choices. Moreover, psychologists in several jurisdictions in the United States (e.g., Louisiana, New Mexico, Idaho, Illinois, and Iowa) and in Europe (e.g., The Netherlands) can pursue modest additional training to obtain prescription privileges. Perhaps most importantly, while psychologists generally are not trained in medication management, they are trained in research design, methodology, and clinical practice. As such, it is the view of the authors that clinical psychologists are not passive spectators in the debate as to whether SSRIs are a valid and ethical treatment modality, they are active participants.

To this end we pose the question: If the serotonin hypothesis is dying (or perhaps already dead), how will clinical psychologists respond? We would like to propose a few avenues that might lead to favorable outcomes. First, as in many treatment approaches, awareness is the first step. We suggest active discussion within the clinical psychology community regarding the impacts of the downfall of the serotonin hypothesis and potential ways forward. Such discussions could take place in the form of special issues within clinical psychology journals, such as *Psychological Science, Journal of Consulting and Clinical Psychology*, and *Clinical Psychological Science*. These could also take the form of panel discussions at popular clinical psychology conferences (e.g., the conventions for the *Association for Contextual Behavioral Science* and *Association for Behavioral and Cognitive Therapies*), and general discussions (as is already occurring) on professional listservs. Psychologists may also consider harnessing social media and other digital platforms to disseminate evidence-based information to lay audiences. Social media not only serves as a promising avenue for connecting scientists and clinicians to the public, but is also a powerful tool to combat the online spread of false information.

These conversations should also be extended to the classroom. Most graduate training programs, and specialized undergraduate courses (e.g., abnormal psychology, biological psychology), discuss aspects of the serotonin hypothesis in varying degrees. It is important that these conversations be contextualized given the current body of evidence as synthesized by researchers such as Moncrieff et al. (2022). Indeed, it is not uncommon for lay persons (including graduate students) to discuss the serotonin hypothesis in conversation as if it were a scientific law. By adjusting how we educate future psychologists, we can curtail the tide of misinformation.

Second, as clinicians, we may need to adjust how we discuss SSRI medications with our clients. This involves being aware of the current state of the literature and being honest about expectations. This is particularly germane for clients who may request our opinions regarding medication use, especially those experiencing treatment resistance. Consistent with our first recommendation, it would be beneficial for clinicians to document their experiences of such conversations and then share them on professional platforms (e.g., qualitative publications) for broader discussion. To be clear, we do not advocate for any current clients to discontinue SSRIs without proper consultation. Indeed, even if serotonin re-uptake inhibition is not curing depressive symptoms, withdrawal associated with SSRI discontinuation may disrupt neural systems causing harmful side effects (Cosci and Chouinard, 2020; Massabki and Abi-Jaoude, 2021).

Third, we need to adjust how we consult with prescribing physicians. We have permission to collaboratively process risks and benefits of SSRI prescriptions in light of the latest research with physicians. As noted, while most clinical psychologists are not trained in medicine, we are trained in research methodology. In this context, we have a right to inform prescribers of scientific literature questioning SSRI prescription and are well-positioned to do so. Moreover, we also have the privilege of collaborating with physicians in exploring other evidence-based options aside from pharmacology (e.g., transcranial magnetic stimulation and process-based talk therapies) in the service of optimizing client care. Considering SSRIs are often prescribed for a wide variety of mental health symptoms (e.g., anxiety), psychologists with and without prescription privileges are encouraged to review empirical evidence for the efficacy of SSRIs in other psychiatric conditions in addition to affective/mood disorders. Indeed, the entire concept of “treatment resistance” might need to be reconsidered in light of the research showing that one of the first line “treatments” is probably not valid.

Fourth, ongoing problems with latent disease classification (e.g., the Diagnostic and Statistical Manual) has led funders to support more biologically-based programs of research (most notably the Research Domain Criteria; Insel et al., 2010). The demise of the serotonin hypothesis is an indicator that purely biological explanations may not be satisfactory in determining mental illness etiology as presently conceptualized. Clinical scientists are encouraged to postulate/explore integrative programs of research that move away from reductionist models of latent constructs. We encourage researchers to explore process-based, biopsychosocial, and transtheoretical models of disorder etiology. Furthermore, we encourage more basic phenomenological research into affective disorders, and more broadly, mental health problems. Modern technology and statistical software have simplified difficult research designs, such as ecological momentary assessment and qualitative research. We encourage federal funders to consider such designs more often. These recommendations coincide with the rise of process-based research. With additional phenomenological research, integrative models can be developed, tested, and refined with greater breadth and depth.

Altogether, it is hoped that how clinical psychologists respond to these serotonin hypothesis developments will reduce needless human suffering. The serotonin hypothesis represents a historical milestone much the same way Freudian models of depression were beyond paranormal theories. In moving away from the serotonin hypothesis, we have the opportunity to find a more valid approach to affective disorder etiology, treatment, and prevention. By embracing the opportunities associated with this paradigm shift, clinical psychologists will continue to play an essential role in helping our communities, while also defining the programs of research of the future.

## Author contributions

Both authors agree to be accountable for ensuring integrity and accuracy of this work. Both authors contributed to the article and approved the submitted version.

## Funding

We thank the Texas Tech University Department of Psychological Sciences for supporting open-access costs.

## Conflict of interest

The authors declare that the research was conducted in the absence of any commercial or financial relationships that could be construed as a potential conflict of interest.

## Publisher's note

All claims expressed in this article are solely those of the authors and do not necessarily represent those of their affiliated organizations, or those of the publisher, the editors and the reviewers. Any product that may be evaluated in this article, or claim that may be made by its manufacturer, is not guaranteed or endorsed by the publisher.
